# Breastmilk Is a Novel Source of Stem Cells with Multilineage Differentiation Potential

**DOI:** 10.1002/stem.1188

**Published:** 2012-08-03

**Authors:** Foteini Hassiotou, Adriana Beltran, Ellen Chetwynd, Alison M Stuebe, Alecia-Jane Twigger, Philipp Metzger, Naomi Trengove, Ching Tat Lai, Luis Filgueira, Pilar Blancafort, Peter E Hartmann

**Affiliations:** a School of Chemistry and Biochemistry, The University of Western AustraliaCrawley, Western Australia, Australia; b School of Anatomy, Physiology and Human Biology, The University of Western AustraliaCrawley, Western Australia, Australia; c Department of Pharmacology, School of Medicine, University of North CarolinaChapel Hill, North Carolina, USA; d Department of Obstetrics and Gynecology, Division of Maternal-Fetal Medicine, School of Medicine, University of North CarolinaChapel Hill, North Carolina, USA; e Institute of Biochemistry and Molecular Biology, ZBMZ, University FreiburgGermany

**Keywords:** Breastmilk, Mammary gland, Transcription factors, Stem cell, Differentiation, Multipotency

## Abstract

The mammary gland undergoes significant remodeling during pregnancy and lactation, which is fuelled by controlled mammary stem cell (MaSC) proliferation. The scarcity of human lactating breast tissue specimens and the low numbers and quiescent state of MaSCs in the resting breast have hindered understanding of both normal MaSC dynamics and the molecular determinants that drive their aberrant self-renewal in breast cancer. Here, we demonstrate that human breastmilk contains stem cells (hBSCs) with multilineage properties. Breastmilk cells from different donors displayed variable expression of pluripotency genes normally found in human embryonic stem cells (hESCs). These genes included the transcription factors (TFs) OCT4, SOX2, NANOG, known to constitute the core self-renewal circuitry of hESCs. When cultured in the presence of mouse embryonic feeder fibroblasts, a population of hBSCs exhibited an encapsulated ESC-like colony morphology and phenotype and could be passaged in secondary and tertiary clonogenic cultures. While self-renewal TFs were found silenced in the normal resting epithelium, they were dramatically upregulated in breastmilk cells cultured in 3D spheroid conditions. Furthermore, hBSCs differentiated in vitro into cell lineages from all three germ layers. These findings provide evidence that breastmilk represents a novel and noninvasive source of patient-specific stem cells with multilineage potential and establish a method for expansion of these cells in culture. They also highlight the potential of these cells to be used as novel models to understand adult stem cell plasticity and breast cancer, with potential use in bioengineering and tissue regeneration. Stem Cells*2012;30:2164–2174*

## INTRODUCTION

Embryonic stem cells (ESCs) derive from the inner mass of mammalian blastocysts and have the ability to self-renew and differentiate into all body cell types [1]. Self-renewing stem cells also exist in adult tissues buffered within specialized niches. They escape their quiescent state in response to developmental and physical cues in order to maintain homeostasis during tissue turnover or injury [2]. Initially it was thought that adult stem cells have limited differentiation potential restricted to the tissue/organ of origin. However, recent evidence has suggested that certain cell subpopulations within adult stem cell compartments are capable of in vitro and in vivo differentiation into cell types outside their dermal origin under specific microenvironments [3–7].

In the mammary gland, bipotent stem cells (MaSCs) capable of differentiation toward the two main mammary epithelial lineages (myoepithelial and luminal) as well as a hierarchy of progenitor cells are known to exist in the epithelium [8–15]. The existence of stem cells in this organ was first postulated based on the capacity of the mammary epithelium to significantly expand and regress in a repeated fashion throughout adult life [16]. In accordance with this, MaSCs exist in a quiescent state and in scarce numbers in the resting breast, but are activated during pregnancy and lactation, undergoing a controlled program of proliferation, differentiation, and apoptosis stimulated by hormonally-driven cues [17–19]. In addition to their physiological role during pregnancy and lactation, increasing evidence implicates MaSCs in the onset and progression of invasive breast carcinomas with metastatic features and propensity toward both mesodermal (bone) and endodermal (lung) organs [20, 21]. These findings suggest that the mammary gland harbors a stem cell population with unique self-renewal capabilities and a potential plasticity reflected both in normal development and in aberrant conditions of the breast. To date, the molecular determinants and regulators of MaSC normal and aberrant self-renewal and plasticity are unknown due to limited understanding of normal MaSC biology and the absence of cell culture model systems to propagate and characterize these cells. Progress has been hindered by the scarcity and quiescent state of MaSCs in the resting breast and the extremely limited availability of human lactating breast tissues, which contain activated MaSCs in greater numbers [15, 22].

Previously, we demonstrated that the activated MaSC pool of the human lactating breast can be accessed noninvasively via expressed breastmilk [23–26]. Stem cells and differentiated cells from the lactating epithelium enter breastmilk either through cell migration and turnover and/or as a consequence of the mechanical shear forces of breastfeeding. Culture of CD49f^+^ human breastmilk-derived stem cells (hBSCs) in 2D conditions and 3D biomatrices revealed their ability to differentiate toward myoepithelial (CK14^+^) and luminal (CK18^+^) mammary cells, demonstrating properties of MaSCs [24–26].

In this study, we sought to examine regulators of the self-renewal of hBSCs and their plasticity and potential to differentiate toward cell types outside the mammary lineage. A novel stem cell population was identified in breastmilk, expressing an array of ESC-associated genes, including the transcription factors (TFs) OCT4, SOX2, NANOG, and KLF4. In situ histological examination of rare human lactating breast tissue specimens revealed the origin and localization of this hBSC population in the lactating breast epithelium, being almost absent in normal resting breast tissue biopsies. Upon coculture with mouse embryonic feeder fibroblasts (MEFs) in ESC growth conditions, hBSCs exhibited an encapsulated ESC-like colony morphology and phenotype. Moreover, under differentiation conditions hBSCs differentiated into cell types from all three germ layers. These findings demonstrate that the normal lactating breast contains a cell population expressing self-renewal ESC TFs. This, together with the previously reported upregulation of these TFs in breast and ovarian malignancies [27–32], implicates them both in the normal and aberrant expansion of these organs. The presence of these cells in breastmilk highlights it as a novel and noninvasive source of multipotent patient-specific stem cells, with the potential for application in regenerative medicine and in studies of molecular determinants of cancer.

## MATERIALS AND METHODS

### Breastmilk Sample Collection

The study was approved by the Human Research Ethics Committee of The University of Western Australia and the institutional review board (IRB) of the University of North Carolina at Chapel Hill, and all participants provided informed written consent. Healthy breastfeeding women (>70) were recruited in Australia and USA, covering a wide range of lactation stages, from month 1 to year 5, through one or multiple children. Pump-expressed mature breastmilk (5–200 ml) was obtained from each participant and was transported to the laboratory immediately upon expression under aseptic conditions.

### Breastmilk Cell Isolation

Breastmilk was diluted with equal volume of sterile phosphate buffered saline (PBS) (pH 7.4, Gibco, Grand Island, NY, http://www.invitrogen.com) and centrifuged at 805*g* for 20 minutes at 20°C. The fat layer and liquid part skim milk were removed, and the cell pellet was washed three times in PBS and resuspended in 7% fetal bovine serum (FBS, Certified, Invitrogen, Carlsbad, CA, http://www.invitrogen.com) in PBS (blocking buffer). The total cell concentration and viability of each sample were determined with a Neubauer hemocytometer by Trypan Blue exclusion.

### Cell Culture

The human embryonic stem cell line H7 (WiCell via the Human Embryonic Core at UNC, Madison, WI, http://www.wicell.org) was maintained at 37°C and 5% CO_2_ in hESC medium containing Dulbecco's modified Eagle's medium (DMEM)/F12 (Gibco), 20% knockout serum replacement (Invitrogen), 5 ng/ml human basic fibroblast growth factor (Sigma-Aldrich, St. Louis, MO, http://www.sigmaaldrich.com), 100 μM nonessential amino acids (NEAA, Invitrogen), 100 μM β-mercaptoethanol (Sigma-Aldrich), 5% antibiotic/antimycotic (Invitrogen), and 2 μL/ml fungizone (Invitrogen). Mammary epithelial cells isolated from resting breast mammoplasties were maintained in human mammary epithelial cell (HuMEC) complete medium (Invitrogen). These cells were derived from normal resting breast tissue procured from the Tissue Procurement Core Facility of the University of North Carolina Lineberger Comprehensive Cancer Center in accordance with approved IRB protocols (Biomedical IRB, study 09-0777). Primary neonatal human fibroblasts were cultured in DMEM (Gibco) supplemented with 10% FBS (Invitrogen) and 1% antibiotic/antimycotic. Breastmilk cells were seeded in gelatin-coated plates on MEFs in MEF-conditioned hESC medium at densities ranging from 5 × 10^5^ to 5 × 10^6^ per 35 mm dish and incubated at 37°C and 5% CO_2_, with daily media changes after day 5. For secondary and tertiary feeder culture, single colonies were individually picked and transferred to new plates in appropriate volume of fresh medium in a split ratio of 1:2. For spheroid culture, breastmilk cells were seeded in ultra-low binding plates (Co-Star, Corning, Tewksbury, USA, http://www.corning.com2174) in MammoCult medium (Stem Cell Technologies, Vancouver, BC, Canada, http://www.stemcell.com) supplemented with 3% antibiotic/antimycotic and 2 μL/ml fungizone. For passaging of adherent colonies or spheroids in feeder-free conditions, the cells were trypsinized (Gibco) for 5 minutes at 37°C and split 1:3.

### Flow Cytometry

**Figure 1:**
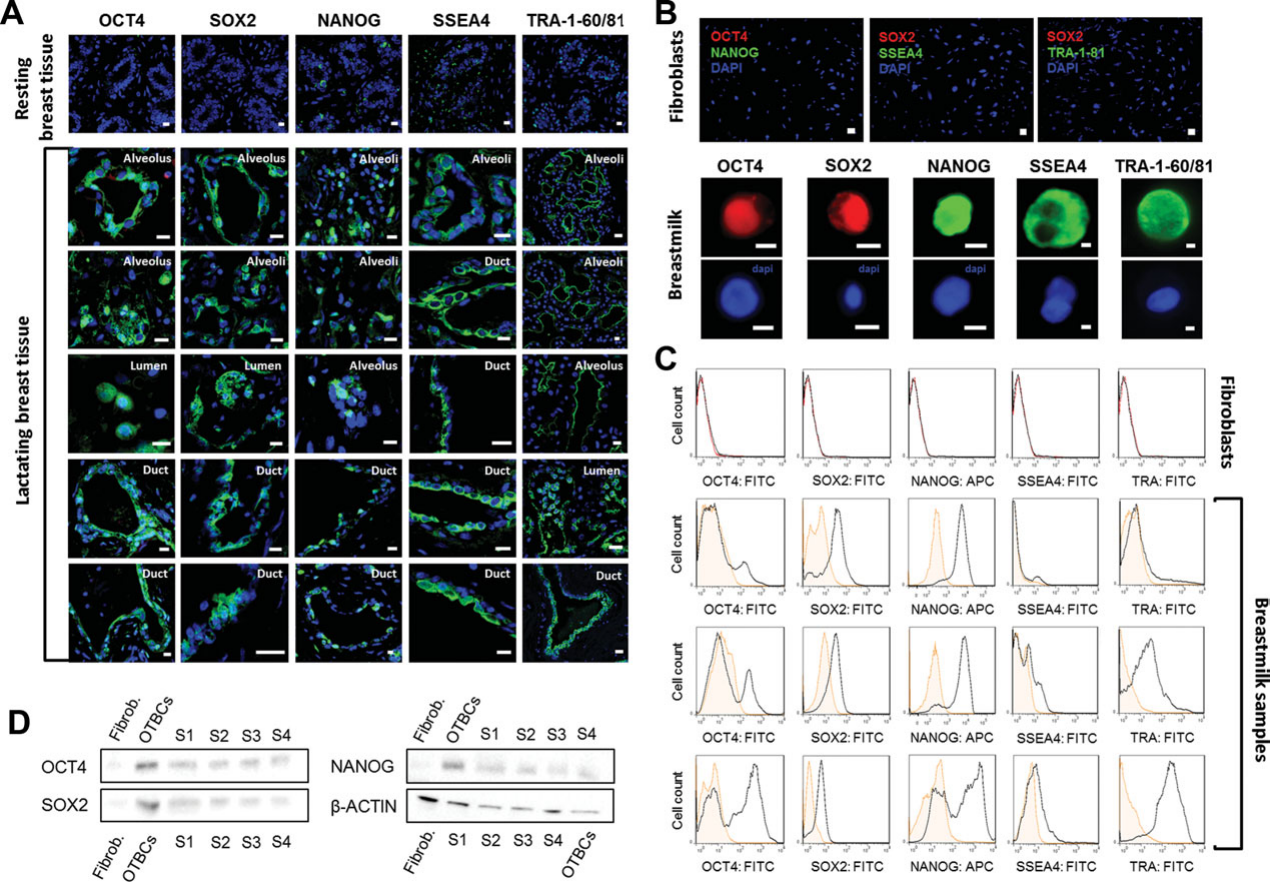
Human breastmilk cells from the lactating mammary epithelium express embryonic stem cell (ESC) genes. (**A**): Immunostaining of normal human resting and lactating breast tissues for expression of ESC genes (OCT4, SOX2, NANOG, SSEA4, and TRA-1-60/81). The absence or minimal expression of ESC genes in the resting breast epithelium is contrasted by the presence of positively stained cells in the alveolar and ductal epithelium of the lactating breast. Positive cells were also observed in the alveolar/ductal lumen, suggesting their presence in breastmilk. Nuclei were stained with 4′,6-diamidino-2-phenylindole (DAPI) (blue). Scale bars = 10 μm. (**B**): Ex vivo immunostaining of freshly isolated breastmilk cells for expression of ESC genes. The first three micrographs show negative control cells (human fibroblasts; scale bars = 50 μm). Nuclei were stained with Hoechst 33342 (blue). Scale bars = 2.5 μm. (**C**): Fluorescence-activated cell sorting (FACS) analysis of freshly isolated breastmilk cells for expression of ESC genes. The first panel shows negative control cells (human fibroblasts). For each marker, examples of FACS breastmilk cell expression profiles from three participants are shown. (**D**): Western blotting showing expression of OCT4, SOX2, and NANOG by freshly isolated breastmilk cells. β-Actin was used as a housekeeping gene. -Fibrob.: human fibroblasts (negative control); OTBCs: OTBC-L1 (positive control); S1–S4 breastmilk samples. Abbreviations: APC, allophycocyanin; OTBC, OCT4-transduced breast cells; FITC, fluorescein isothiocyanate.

Antibodies against ESC markers were standardized using human fibroblasts as negative control (Fig. 1) and were shown to recognize their target proteins by fluorescence-activated cell sorting (FACS) (Stemgent, USA, https://www.stemgent.com/; Santa Cruz Biotechnology, Santa Cruz, CA, http://www.scbt.com). Freshly isolated breastmilk cells were incubated in primary antibody (Supporting Information Table S1) for 30 minutes at 4°C, followed by washes and secondary antibody incubation (AlexaFluor 488 or 647 nm, Invitrogen) for 30 minutes at 4°C at 1:300, and finally washed and resuspended in fixative (1% paraformaraldehyde/0.7% sucrose in PBS). For surface markers, all incubations and washes were done in blocking buffer, while for intracellular markers in 0.05% Tween-20 in PBS after initial cell fixation. Cells were also incubated with live/dead fixable cell stain (Invitrogen) according to the manufacturer's instructions and only the live cells were considered for data analyses. Appropriate negative internal controls were used. Data acquisition was done with a FACS Calibur Flow Cytometer (Becton Dickinson, Franklin Lakes, NJ, http://www.bd.com) and data analysis with FlowJo.

### Quantitative Real-Time Polymerase Chain Reaction

Total RNA from freshly isolated breastmilk cells, harvested hBSC-derived spheroids, hESC H7 cell line, mammary epithelial cells derived from resting tissue mammoplasties, and human primary neonatal fibroblasts was extracted with an RNeasy extraction kit (Qiagen, Valencia, CA, http://www1.qiagen.com) following the instructions of the manufacturer. Total RNA was reverse-transcribed using the high-capacity cDNA archive kit (Applied Biosystems, Carlsbad, CA, http://www.appliedbiosystems.com) plus RNase inhibitor (Applied Biosystems). Gene transcription was quantified by quantitative real-time polymerase chain reaction (qRT-PCR) using hydrolytic probes (Taqman, Applied Biosystems; Supporting Information Table S2) with the 7500 Fast RT-PCR system (Applied Biosystems). Each sample was measured in triplicates or in few cases in duplicates when the extracted RNA was not adequate. Fold change in gene expression for each sample and experimental condition was calculated as 2^Ct(control) − Ct(sample)^ ± SD.

### Western Blotting

Breastmilk cells, human fibroblasts (negative control), and OTBCs (OCT4-transduced breast cells) [33] (positive control) were lysed in RIPA buffer for 30 minutes on ice. Protein was quantified using the Micro BCA protein kit (Thermo Scientific, USA, http://www.thermoscientific.com). Protein denaturation was done for 5 minutes at 95°C. 50 μg of protein was loaded into Biorad TGX stain-free gels, which were run at 300 V for 30 minutes. Protein was transferred onto membranes using a Trans-Blot Turbo System (Biorad). Membranes were washed in 0.05% TBS-T, stained with Ponceau red for 5 minutes, destained with dH_2_O, and washed in 0.05% TBS-T. Membranes were blocked using 5% nonfat cow's skim milk for 1 hour, incubated with primary antibodies (Supporting Information Table S1) overnight at 4°C, washed in 0.05% TBS-T, and incubated with secondary antibodies with 1:10,000 Streptoactin for 1 hour at room temperature. Subsequently, membranes were washed in 0.05% TBS-T before incubation in Chemiluminescence Crescendo (Millipore) for 5 minutes. Imaging was done using a Chemi-Doc MP (Biorad).

Culture supernatants from breastmilk cells cultured under mammary differentiation conditions were harvested on weeks 2–4 of culture and examined for the presence of α-lactalbumin and lactoferrin. Fresh medium and human breastmilk/purified proteins were used as negative and positive controls, respectively. 200 μg of total protein from the culture supernatants was loaded per well of a 10% polyacrylamide gel and run under SDS/PAGE denaturing conditions. Proteins were transferred to a nitrocellulose membrane and blocked for 1 hour in 5% cow's skim milk. Antibodies were applied at the concentrations listed in Supporting Information Table S1. Membranes were then developed (ECL-Plus, GE Healthcare, Piscataway, NJ) and visualized by autoradiography.

### In Vitro Differentiation

To examine the ability of hBSCs to spontaneously differentiate, breastmilk cells were initially grown as spheroids (see above). By day 4–7, some cells had attached. The remaining spheroids were transferred into new wells where adherent cells appeared in 1–2 days. Both the initial and subsequent attached cells were cultured for another 2–3 weeks, with media changes every 3–5 days. For directed differentiation, primary and first- to third-passage breastmilk cell cultures were incubated in differentiation media at 37°C and 5% CO_2_ for 3–4 weeks. For mammary differentiation, cells were incubated in Roswell Park Memorial Institute medium (RPMI) 1640 with l-glutamine (Invitrogen) supplemented with 20% FBS, 4 μg/ml insulin (Invitrogen), 20 ng/ml epidermal growth factor (EGF) (Invitrogen), 0.5 μg/ml hydrocortisone (Sigma-Aldrich), 5% antibiotic-antimycotic, and 2 μL/ml fungizone. For osteoblastic and adipogenic differentiation, cells were incubated in NH OsteoDiff or NH Adipodiff medium, respectively (Miltenyi Biotec, Bergisch Gladbach, Germany, http://www.miltenyibiotec.com). For chondrocyte differentiation, cells were incubated for 3–4 weeks in StemPro Chondrogenesis Differentiation medium (Life Technologies, Rockville, MD, http://www.lifetech.com). For neuronal differentiation, cells were incubated in RPMI 1640 with l-glutamine, supplemented with 20% FBS, 4 μg/ml insulin, 20 ng/ml EGF, 0.5 μg/ml hydrocortisone, 5% antibiotic-antimycotic, 2 μL/ml fungizone, 20 ng/ml prolactin (Peprotech, Rocky Hill, NJ, http://www.peprotech.com), and MACS Supplement B27 (Miltenyi Biotec). For hepatocyte differentiation, cells were cultured in Williams' E basic medium supplemented with 10% FBS, 2.5 μg/ml fungizone, 5% antibiotic/antimycotic, 2 mM glutamine (Sigma-Aldrich), 6.25 μg/ml ITS (insulin, human transferrin, and selenious acid supplement) (Invitrogen), 20 ng/ml EGF (BD Biosciences), 10 mM nicotinamide, and 10^−7^ M dexamethasone. For pancreatic differentiation, cells were first incubated for 2 days in high-glucose DMEM supplemented with 10% FBS, 2.5 μg/ml fungizone, 5% antibiotic/antimycotic, 2 mM glutamine, 6.25 μg/ml ITS, 20 ng/ml EGF, 50 ng/ml activin A (Sigma-Aldrich), and 10^−6^ mol/L retinoic acid (Sigma-Aldrich). For the next 9–12 days, cells were incubated in DMEM/F12 supplemented with 10% FBS, 2.5 μg/ml fungizone, 5% antibiotic/antimycotic, 2 mM glutamine, 6.25 μg/ml ITS, 20 ng/ml EGF, 10 mM nicotinamide, 10^−7^ M dexamethasone, and 300 nmol/L indolactam V (Sigma-Aldrich). For the final 2–4 days of differentiation, the above medium was supplemented with 50 ng/ml activin A. For cardiomyocyte differentiation, cells were cultured in DMEM/F12 supplemented with 20% FBS, 5% horse serum (Invitrogen), 2 mM l-glutamine, 0.1 M NEAA, 3 mM sodium pyruvate (Invitrogen), 1 μg/ml insulin, 2.5 μg/ml fungizone, and 5% antibiotic/antimycotic.

### Mammary Tissues for Immunohistochemistry

Lactating and resting mammary tissue sections (5 μm-thick, derived from three different tissue blocks for each type) were prepared from normal human biopsied formalin-fixed and paraffin-embedded tissue currently available in the tissue archive of the School of Anatomy, Physiology and Human Biology, The University of Western Australia.

### Immunostaining

Antibodies for ESC markers were standardized using human fibroblasts as negative control cells (Fig. 1). All antibodies used were shown to recognize their target proteins by immunocytochemistry (Supporting Information Table S1). Part of the breastmilk cell suspensions was immediately fixed in 1.5% paraformaraldehyde/0.7% sucrose in PBS and cytospins were generated using a Shandon Cytospin 3 centrifuge at 40.7*g* for 4 minutes. Primary antibodies (Supporting Information Table S1) were applied in blocking buffer for 1 hour at room temperature in a humid chamber, after a 5-minute permeabilization with 0.05% Tween-20 in PBS for intracellular markers. Cytospins were washed, incubated with secondary antibody (AlexaFluor 488, 546, or 555 nm, Invitrogen) in blocking buffer at 1:300 and DAPI (1:100, Roche) for 30 minutes, washed and mounted in DakoCytomation Fluor. mounting medium. Adherent cells grown on plastic plates were fixed as above, permeabilized in 0.1% Triton X-100 in PBS for 30 minutes, incubated with primary and secondary antibodies as above, washed and resuspended in fixative. Spheroids were fixed in 3% formaraldehyde in PBS, permeabilized in 0.1% PBS-Triton X-100 for 20 minutes, washed and stained with primary and secondary antibodies (as above) for 6 hours and 2 hours, respectively. Breast tissue sections were rehydrated in deionized water overnight at 4°C, followed by a 10-minute incubation in PBS, permeabilization in 0.1% PBS-Triton-X 100 for 15 minutes in a humid chamber, and a wash in PBS prior to overnight incubation at room temperature with primary antibody (Supporting Information Table S1) in blocking buffer. The sections were then washed in PBS and incubated with secondary antibody (as above) for 2 hours, followed by a final wash and mounting. Appropriate negative controls (with no primary antibody incubation) were used to standardize imaging, which was done using a Nikon A1Si Confocal microscope, an Olympus TH4-200 inverted optical microscope, a Nikon Eclipse Ti inverted optical microscope or a Leica DMIRB Inverted Fluorescence/DIC microscope.

### Teratoma Formation Assay

The protocol was approved by the Animal Research Ethics Committees of The University of Western Australia and the University of North Carolina at Chapel Hill. Freshly isolated breastmilk cells (3 × 10^6^–32 × 10^6^) or spheroid-derived breastmilk cells (2 × 10^5^–10^7^) were suspended in 30% Matrigel (BD Biosciences) in PBS and injected subcutaneously into dorsal flanks of 15 severe combined immunodeficient (SCID) mice. Nine weeks after injection, mice were examined for tumor formation. p86-OTBC-L1 (OCT4-tranduced breast cells) [33] were used as positive control.

### Statistical Analysis

All statistical analyses, including descriptive statistics, graphical exploration of the data, and Student's *t*-tests for statistical significance were performed in Microsoft Excel and in R 2.9.0 one [34]. The results are presented as mean ± SEM as indicated in figure legends, and *p* values are shown in the figures.

## RESULTS

### Human Breastmilk Contains a Population of Cells Expressing ESC Genes

We set out to examine molecular determinants of self-renewal and indicators of plasticity in MaSCs in human breast tissue specimens by immunofluorescence microscopy (IF). Normal human resting and lactating breast biopsies were examined for expression of genes that constitute the core circuitry of self-renewal in hESCs. No or minimal expression of OCT4, SOX2, NANOG, SSEA4, and TRA-1-60/TRA-1-81 was observed in resting mammary tissues (Fig. 1A). By contrast, ESC-associated genes were clearly expressed in normal lactating mammary tissues, where distinct patterns of gene expression were recorded (Fig. 1A). The TFs OCT4, SOX2, and NANOG were expressed both in the luminal and basal epithelial layers in the alveoli and ducts of the lactating epithelium (Fig. 1A). Differences were observed in the distribution and level of ESC gene expression between different ducts and alveolar batches, confirming a previously reported developmental heterogeneicity between different epithelial compartments within the same breast tissue [35]. Moreover, even within a duct or alveolus expression differences were evident among different epithelial cells, ranging from absence of expression, minimal cytoplasmic expression, clear nuclear expression, and mixed cytoplasmic and nuclear expression within a cell (Fig. 1B). For all three TFs, the highest expression level was observed in the myoepithelial layer compared to the luminal layer both in ducts and alveoli. SSEA4 was more irregularly expressed in both ducts and alveoli (Fig. 1A). TRA-1-60 and TRA-1-81 were restricted to the luminal cell surface in alveoli but were more irregularly expressed in ducts. Coexpression of these markers was observed in cell subpopulations in the ductal and alveolar zones. Cells positive for these markers were also captured in the lumen of ducts and alveoli (Fig. 1A), suggesting their presence in breastmilk.

**Figure 2:**
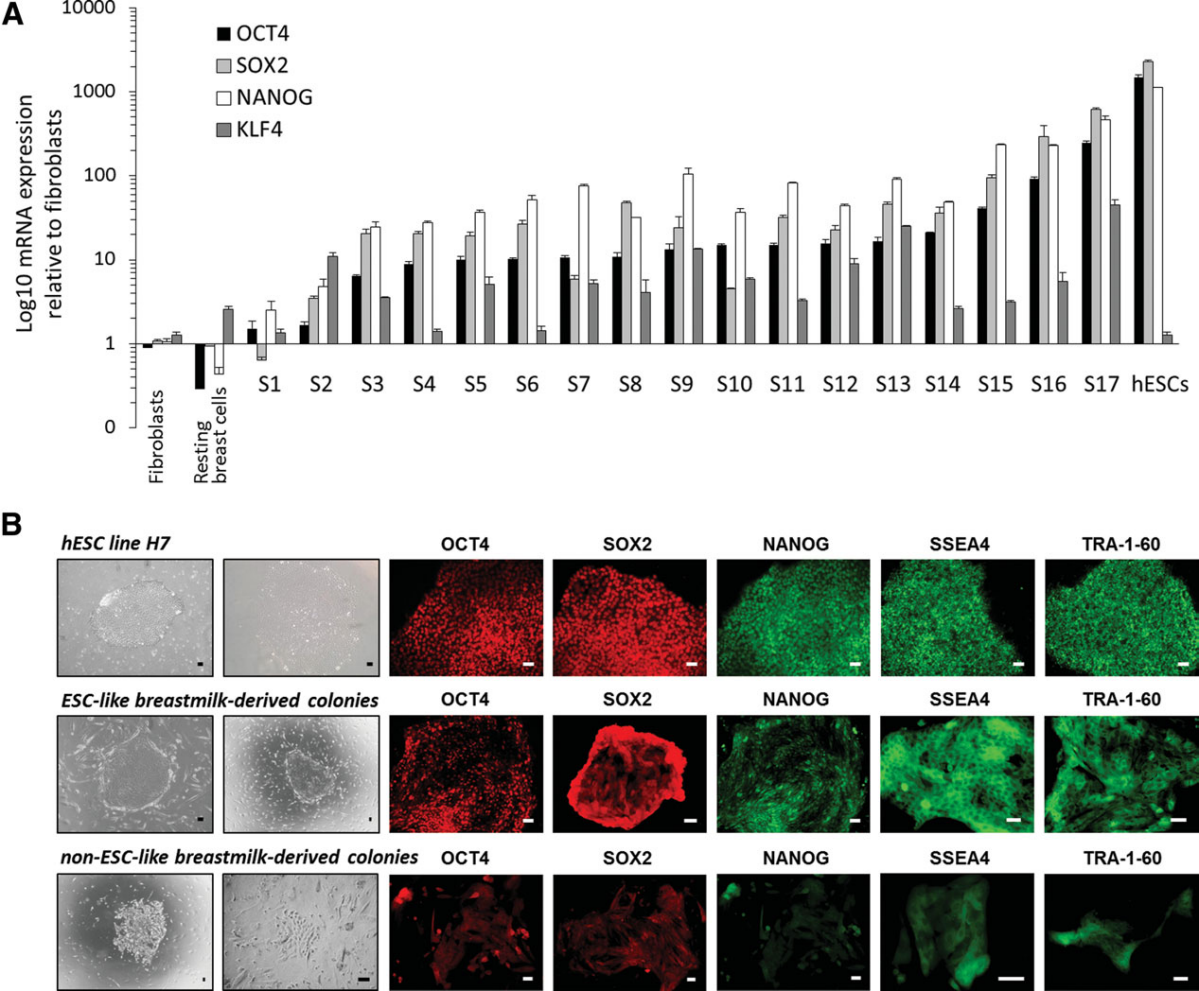
Breastmilk stem cells expressing ESC genes display clonogeneicity and ESC-like colony morphology and phenotype. (**A**): Quantitative real-time polymerase chain reaction (PCR) assay for expression of OCT4, SOX2, NANOG, and KLF4 by human breastmilk cells in 17 breastmilk samples (S1–S17), and comparison with human fibroblasts, mammary epithelial cells isolated from resting breast mammoplasties, and hESCs. Individual PCR reactions were normalized against internal controls (GAPDH) and plotted relative to the expression level of the fibroblasts. Bars represent the mean ± SEM. (**B**): Coculture of human breastmilk-derived stem cells (hBSCs) with mouse embryonic feeder fibroblasts in hESC medium resulted in formation of two distinct types of adherent colonies: ESC-like breastmilk-derived colonies (second panel) with morphology similar to hESCs (cell line H7, first panel); and non-ESC-like breastmilk-derived colonies (third panel) with various morphologies. All ESC-like hBSC colonies expressed OCT4, SOX2, NANOG, SSEA4, SSEA3, and TRA-1-60, similar to hESC line H7. Most non-ESC-like colonies expressed these genes at low levels, if at all. Scale bars = 50 μm. Abbreviation: hESCs, human embryonic stem cells.

To noninvasively access this enriched ESC-gene+ cell pool from the lactating breast, we used freshly expressed breastmilk. Total breastmilk cells were isolated and examined for ex vivo expression of ESC-associated genes at the mRNA and protein levels, using IF (Fig. 1B), FACS (Fig. 1C), Western blotting (WB) (Fig. 1D), and qRT-PCR (Fig. 2A). All antibodies and probes were first standardized using human fibroblasts as negative control (Fig. 1B, 1C). A clear nuclear, and sometimes perinuclear or cytoplasmic localization of OCT4, SOX2, and NANOG was observed using IF in breastmilk cell subpopulations, whereas SSEA4 and TRA-1-60/TRA-1-81 were localized on the cell surface (Fig. 1B). An analytic gating strategy was developed for quantification of protein expression by FACS, which included exclusion of interference by dead cells and/or fat globules (Supporting Information Fig. S1a). A remarkable variation in expression levels was observed by FACS among the breastmilk cell preparations examined, ranging 4%–96% of total cells for OCT4, SOX2, and NANOG, and 0.1%–93% for the surface antigens SSEA4 and TRA-1-60/TRA-1-81 (Supporting Information Table S3; Fig. S1b). Among these genes, TRA-1-60/TRA-1-81 and NANOG were the most highly expressed, followed by OCT4 (Supporting Information Fig. S1b). Coexpression of these genes by single cells was demonstrated both with IF and FACS (Supporting Information Fig. S2). Similar to the variation at the protein level, which was confirmed also by WB (Fig. 1D), a wide variation was observed in mRNA expression levels among samples from different women (Fig. 2A). OCT4, SOX2, and NANOG mRNA expression was lower in hBSCs compared to the hESC line H7 but significantly higher than that of human fibroblasts and cells derived from resting breast mammoplasties (*p* < 0.0001) (Fig. 2A). Among the breastmilk samples analyzed, the highest level of ESC gene mRNA expression was observed in a participant who was breastfeeding and was concurrently pregnant to her next child. hBSCs expressed NANOG mRNA at higher levels compared to OCT4 and SOX2, which was consistent with the results obtained using IF and FACS for protein expression. Interestingly, KLF4 expression by hBSCs was significantly higher than that of hESCs (*p* < 0.0001) (Fig. 2A). In addition to the above ESC-associated genes, hBSCs expressed REX1, hTERT, and GDF3 at variable levels, with some samples showing comparable or slightly higher expression than that of hESCs (Supporting Information Fig. S3). These results established expression of ESC-associated genes at the protein and mRNA levels by a subpopulation of breastmilk cells.

### Clonogeneicity of Breastmilk Stem Cells Expressing ESC Genes

A distinguishing property of hESCs is the formation of flat, compact, and encapsulated colonies in coculture with MEFs in hESC medium [36]. These colonies typically express the pluripotency genes OCT4, SOX2, NANOG, SSEA4, and TRA-1-60/TRA-1-81 (Fig. 2B). To examine the clonogeneicity, morphology, and phenotype of hBSCs in coculture with MEFs and compare it with that of hESCs, cells isolated from freshly expressed breastmilk were cultured with MEFs in hESC medium. A rapid cellular proliferation was observed first in suspension, during which individual cells divided and formed spherical structures. Although expansion in suspension continued, within 4–7 days of plating adherent individual cells and colonies appeared. The suspension cells were removed with the first media change, and the adherent colonies were allowed to expand. Two distinct types of adherent colonies were observed: ESC-like flat, compact, encapsulated colonies with high nucleus:cytoplasm ratio, and non-ESC-like colonies (Fig. 2B). The non-ESC-like colonies had various morphologies, from a mesenchymal-like to an epithelial-like or mixed morphology (Fig. 2B). The formation frequency of the two colony types differed between different breastmilk samples, with 68%–100% (mean 90% ± 3%, *n* = 11) of all colonies displaying the ESC-like morphology. In our hands and for the breastmilk samples tested, the frequency of ESC-like colony forming cells ranged 1 in 15,000 to 1 in 1,750,000 (*n* = 11) (Supporting Information Table S4).

All ESC-like hBSC colonies expressed OCT4, SOX2, NANOG, SSEA4, SSEA3, and TRA-1-60/TRA-1-81, with the TFs being localized primarily in the nucleus (Fig. 2B). Similarly to hESCs, spontaneous differentiation in the center of the ESC-like colonies was occasionally observed, particularly when they were allowed to expand for more than 2 weeks. Most non-ESC-like colonies expressed these genes at low levels, if at all. Based on TF gene expression, three distinct cell types were observed within the non-ESC-like colonies: negative cells, dimly positive cells with TF expression primarily in the cytoplasm, and few smaller round weakly attached cells that were clearly positive in the nucleus (Fig. 2B). SSEA4 and TRA-1-60/TRA-1-81 were expressed at higher levels than the TFs in the non-ESC-like colonies, but at lower levels than in the ESC-like colonies. It must be noted that in addition to colonies, single attached cells that failed to expand in 2D were also observed. Many of these cells expressed ESC-associated markers at high levels. The nature and properties of these cells remain to be established.

ESC-like colonies were passaged in secondary and tertiary feeder cultures, where they generated identical colonies with ESC-like morphology and phenotype. Similar colony formation characteristics were observed when breastmilk cells were cultured in the absence of feeders in gelatin-coated or uncoated adhesion plates (Supporting Information Fig. S4a), although attachment and colony formation success was higher in the presence of feeders. By contrast, ESC-associated TFs were not expressed in cultures derived from mammoplastic reductions of resting breast tissue (Fig. 2A; Supporting Information Fig. S4b). These data suggest that a subpopulation of hBSCs possesses ESC-like features and clonogeneicity.

### 3D Culture of Breastmilk Stem Cells Enriches for Self-Renewal ESC TFs

**Figure 3:**
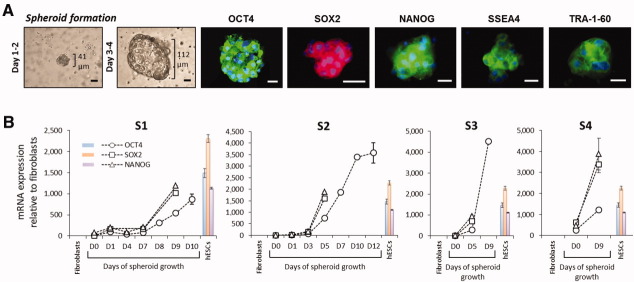
Breastmilk stem cells expressing embryonic stem cell (ESC) genes display self-renewal in 3D culture. (**A**): 3D culture of breastmilk cells and formation of single cell-originated spheroids that expressed ESC genes (OCT4, SOX2, NANOG, SSEA4, and TRA-1-60) and which could be serially passaged, demonstrating self-renewal. Typically, the most rapid increase in spheroid size was observed within the first 1–4 days (first two panels). Nuclei were stained with 4′,6-diamidino-2-phenylindole (DAPI) (blue). Scale bars = 20 μm. (**B**): Time-course of ESC gene expression by breastmilk cells in 3D culture conditions, demonstrating upregulation of ESC genes during the first 12 days of spheroid formation. Quantitative real-time polymerase chain reaction (PCR) assay for expression of OCT4, SOX2, and NANOG in four breastmilk-derived cell samples (S1–S4) from four different participants at day zero (D0 – fresh breastmilk cells prior to culture), and then days 1–12 (D1–D12) of spheroid growth. Each point on the graphs represents the mean ± SEM. Individual PCR reactions were normalized against internal controls (GAPDH) and plotted relative to the expression level of human fibroblasts. For comparison purposes, expression levels of the hESC line H7 are shown in bars (mean ± SEM). More examples are shown in Supporting Information Figure S5.

As we observed a propensity for initial expansion of hBSCs in suspension via spheroid formation (even in adhesion plates), we next examined characteristics of breastmilk cells when cultured in 3D in ultra-low binding plates. These conditions are well known to enrich for the presence of stem and progenitor cells in a cell population [12]. With or without the presence of extracellular matrix (Matrigel), breastmilk cells rapidly formed spheroids, which could be successfully maintained through several passages (Fig. 3A; Supporting Information Fig. S4c). Typically, the most rapid increase in spheroid size was observed within the first 1–4 days (Fig. 3A). The ability of spheroid formation, spheroid sizes, and size increase in the course of culture varied between different breastmilk samples, with smaller magnitude variations observed also between spheroids of the same sample. Expression of ESC-associated genes by breastmilk-derived spheroids was confirmed by IF (Fig. 3A), which also revealed coexpression of these genes (Supporting Information Fig. S4d), and by qRT-PCR (Fig. 3B).

Interestingly, a significant upregulation of self-renewal TFs was observed during spheroid formation that equalled or sometimes exceeded the mRNA expression levels of hESCs. A time-course analysis of OCT4, SOX2, and NANOG mRNA expression from day 1 to day 12 of spheroid formation revealed a stable upregulation of these genes, which typically peaked after day 7, and reached or exceeded the expression levels of hESCs (Fig. 3B). Of note, variations in the extent of upregulation of ESC-associated genes and the day on which expression levels peaked were observed among different breastmilk samples (Supporting Information Fig. S5). Therefore, culture of breastmilk cells in suspension provides a rapid method for expansion of a hBSC population that expresses self-renewal genes associated with ESCs.

### Differentiation of Breastmilk Stem Cells into Cells Originating from the Three Germ Layers

To determine the differentiation potential of hBSCs, we followed three approaches. In hESCs and human induced pluripotent cells (hiPSCs), an initial embryoid body formation in 3D and subsequent cultivation of the embryoid bodies in adherent conditions results in mixed spontaneous differentiation into cells from all three germ layers [36, 37]. Breastmilk cells cultured in adherence plates first formed spheroids and by day 4–7 some cells had attached. After 2–3 weeks, the resultant cultures contained a mixture of adherent cells/colonies with various morphologies, including those of epithelial cells, mesenchymal cells, neural cells, and cobblestone-like cells, which stained positive for corresponding markers (Supporting Information Fig. S6).

**Figure 4:**
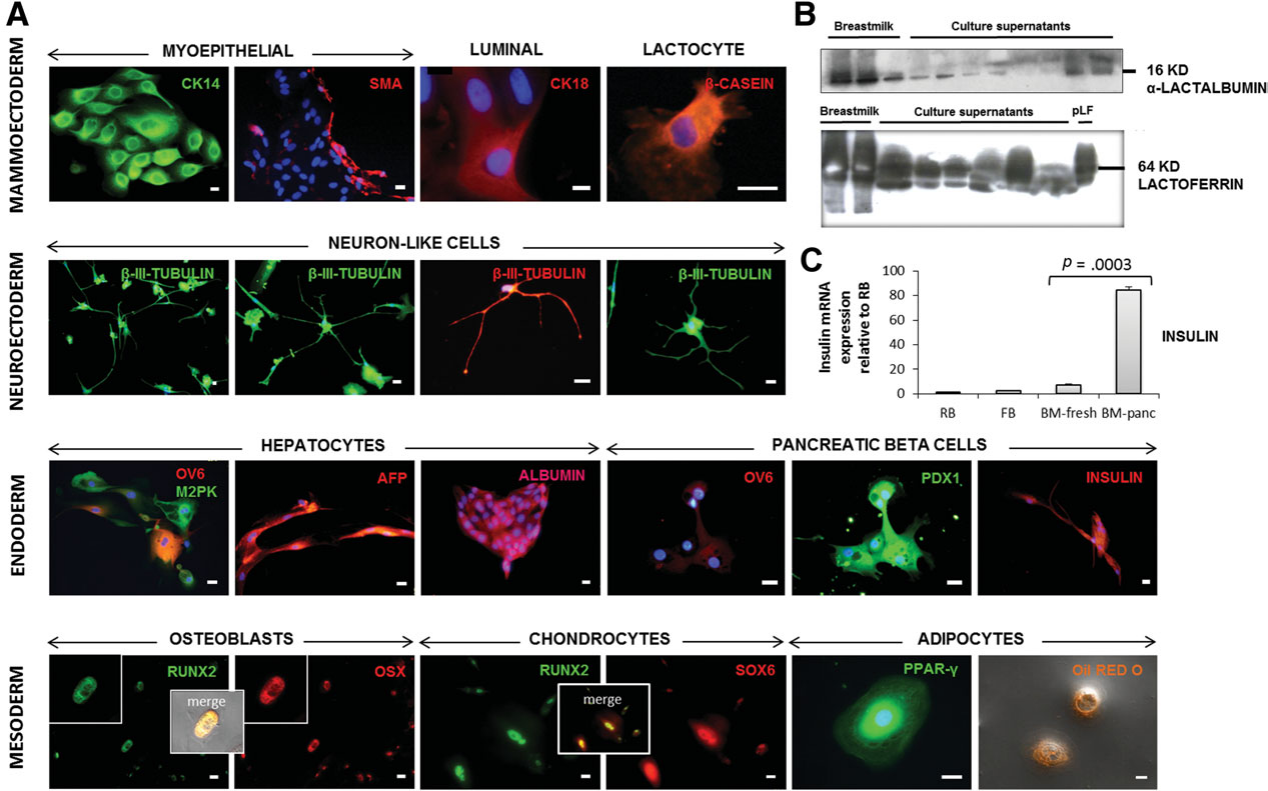
Breastmilk stem cells differentiate into cells of the three germ layers. (**A**): Immunostaining for mammoectodermal, neuroectodermal, endodermal, and mesodermal differentiation under corresponding growth conditions. *Mammary differentiation*: Myoepithelial cells (CK14^+^, SMA^+^) and luminal cells (CK18^+^/CD49f^−^), some of which synthesized milk proteins (β-casein). *Neural differentiation*: Neuronal-like colonies expressing β-III-tubulin. *Hepatocyte differentiation*: Initial upregulation of the endodermal progenitor marker OV6 and subsequent upregulation of the mature hepatocyte markers AFP and M2PK, and the functional hepatocyte marker albumin. *Pancreatic differentiation*: Pancreatic beta cells (PDX1^+^/insulin^+^). *Osteogenic differentiation*: Colonies with the osteoblastic profile (RUNX2^+^/OSX^+^). *Chondrogenic differentiation*: Colonies with the chondrocyte marker profile (RUNX2^+^/SOX6^+^). *Adipogenic differentiation*: PPAR-γ^+^ cells that formed large lipid droplets (oil red O^+^). Nuclei were stained with 4′,6-diamidino-2-phenylindole (DAPI) (blue). Scale bars = 20 μm. (**B**): Western Blot analysis of secreted milk proteins (α-lactalbumin and lactoferrin) in culture supernatants under mammary differentiation conditions. Breastmilk and purified proteins were used as positive controls, and fresh medium as negative control. (**C**): Quantitative real-time polymerase chain reaction (PCR) assay for insulin expression in breastmilk-derived cultures under pancreatic differentiation conditions. Insulin mRNA expression levels in freshly isolated breastmilk cells (BM-fresh) and breastmilk cells cultured under pancreatic conditions (BM-panc) are compared with those of mammary epithelial cells isolated from resting breast mammoplasties (RB) and human fibroblasts (FB) cultured under the same pancreatic differentiation conditions. Individual PCR reactions were normalized against internal controls (GAPDH) and plotted relative to the expression level of RB. Bars represent the mean ± SEM. Abbreviation: AFP, α-fetoprotein.

In a second approach, we examined lineage-directed differentiation of breastmilk cells when cultured in 2D in specific growth media, using methods previously reported for hESCs and hiPSCs [36]. Initially, the potential of differentiation into the two mammary lineages was confirmed. Within the first 2 weeks of culture in mammary differentiation conditions, myoepithelial cells (CK14^+^, SMA^+^) were observed in the attached colonies (Fig. 4A). On week 3, luminal cells (CK18^+^/CD49f^−^) were detected, some of which spontaneously synthesized milk proteins (β-casein, lactoferrin, α-lactalbumin), which were secreted and detected in the culture supernatant (Fig. 4B; Supporting Information Fig. S7). Under neurogenic culture conditions, cells with neuronal morphology and expression of nestin and β-III-tubulin were detected within 2–3 weeks of culture (Fig. 4A). Under osteoblastic conditions, we detected cells with nuclear expression of RUNX2, a TF essential for osteoblastic differentiation (Fig. 4A). Some RUNX2^+^ cells coexpressed OSX, an osteoblast-specific TF required for bone formation (Fig. 4A). Under chondrogenic conditions, cells with the chondrocyte marker profile (RUNX2^+^/SOX6^+^) were identified after 2–3 weeks of culture (Fig. 4A). Under conditions promoting adipogenic differentiation, cells positive for the adipogenic TF PPAR-γ, which developed large lipid droplets (oil red O^+^) were observed (Fig. 4A). Breastmilk cells cultured in hepatocyte differentiation conditions initially upregulated the endodermal progenitor marker OV6, which was subsequently downregulated with concurrent upregulation of the mature hepatocyte markers α-fetoprotein and M2 isoenzyme of pyruvate kinase (M2PK), and the functional hepatocyte marker albumin (Fig. 4A). Under pancreatic differentiation conditions, upregulation of the mature beta cell marker PDX1 was observed, and expression of insulin in the resulting cells was confirmed by IF and qRT-PCR (Fig. 4A, 4C).

In a third approach, directed differentiation in 3D conditions was examined by growing breastmilk-derived spheroids in differentiation media for a longer period. Indeed, in corresponding media, mammospheres producing milk proteins (β-casein), pancreatospheres expressing insulin, and cardiospheres expressing cardiac T troponin were obtained within 3–4 weeks of culture (Supporting Information Fig. S8). In all differentiation inductions, upregulation of the differentiation genes was accompanied by downregulation of ESC-associated genes.

### Teratoma Assay of Breastmilk Cells

To establish whether breastmilk cells are capable of forming teratomas when injected subcutaneously into dorsal flanks of immunodeficient (SCID) mice, 15 mice were injected with freshly isolated total breastmilk cells (3 × 10^6^–32 × 10^6^) or with spheroid-derived breastmilk cells (2 × 10^5^–10^7^). Nine weeks after injection, mice were examined and no tumor formation was observed. By contrast, positive control animals injected with as few as 50 p86-OTBC-L1 (OCT4-tranduced breast cancer cells) showed formation of poorly differentiated tumors [33].

## DISCUSSION

We have shown that a stem cell population with multilineage potential that expresses self-renewal TFs typical of ESCs exists in the human lactating breast and can be noninvasively accessed via breastmilk. This cell population was almost absent from the normal resting breast, suggesting (a) that at least some of these cells may be mobilized from other tissues, and/or (b) that it is remnant from embryonic development in the resting breast, where it is extremely scarce and in a quiescent state. We speculate that pregnancy/lactation-associated hormonal cues activate this cell population via stimulation of a self-renewal program mediated by a marked upregulation of ESC-associated genes.

The lactating mammary epithelium was characterized by a cellular hierarchy reflected in the cellular heterogeneicity observed in breastmilk. Typically, a high, yet variable, percentage of cells expressing the ESC pluripotency TF circuitry was found. Nevertheless, the differing subcellular localization (from cytoplasmic to perinuclear to nuclear) and levels of expression among different cells further reinforce the presence of a cellular hierarchy, from earlier-stage stem cells to committed progenitors, and potential stages in between. Although all these cells express ESC-associated genes at some level, we propose that not all are at the same developmental stage and not all have the same self-renewal and differentiation potential. This was reflected at the much lower frequency of ESC-like colony formation by breastmilk-derived cells in feeder culture. Importantly, these colonies could be serially passaged in secondary and tertiary feeder cultures, demonstrating clonogeneicity. In addition to ESC-like colonies, non-ESC-like colonies were observed, some of which retained low expression of ESC-associated genes. The non-ESC-like colonies displayed varying morphologies, from epithelial to mesenchymal to mixed. Based on these findings, we hypothesize that the non-ESC-like colonies originate from the expansion of more committed progenitors present in breastmilk, suggesting that ESC-associated genes are not completely downregulated in the more committed mammary progenitor cells.

It therefore becomes clear that a wide spectrum of developmental stages and functionalities is represented in breastmilk. Among the more committed progenitor and differentiated cells, breastmilk contains a stem cell population, which shares some properties with hESCs, yet it is not identical to them. Freshly isolated breastmilk cells expressed the core TF network governing pluripotency in hESCs [38]. Interestingly, of the three TFs, NANOG was expressed at higher levels among the breastmilk samples tested. NANOG is the key self-renewal regulator essential for early development and for maintaining the ground-state pluripotency of ESCs [39]. With the exception of KLF4, which was expressed at much higher levels by breastmilk cells compared to hESCs, expression levels of the other ESC genes examined were variable and generally lower than hESCs. This could reflect the heterogeneicity of the breastmilk cellular compartment or the lower expression of the markers by hBSCs, or both, and reinforces the concept of a unique cellular composition of breastmilk. Interestingly, 3D culture conditions enriched for the ESC-gene-expressing hBSCs in a time-dependent manner, with expression levels of ESC-associated genes that equalled or exceeded those of hESCs. This is in accordance with previous studies reporting increased expression of OCT4 during spheroid culture of breast cancer cell lines [40] and of epithelial cells derived from resting breast tissue mammoplasties (Blancafort, unpublished data). However, the increase in OCT4 expression during spheroid culture in both breast cancer cell lines and normal epithelial cells from resting breast tissue is in the order of three to five times, whereas in hBSCs in the order of hundreds to thousands of times, often reaching or exceeding the expression levels of hESCs (Fig. 3B). These findings support the pre-existence in the resting breast of a quiescent stem cell population, which expands during pregnancy and lactation and provide a useful method for in vitro expansion of the ESC-like hBSC population for future purposes.

In addition to ESC gene expression and clonogeneicity, hBSCs demonstrated spontaneous differentiation toward various cell types in vitro and were capable of directed differentiation into cell types of mammary, neuroectodermal, mesodermal, and endodermal origins. The resultant differentiation progressed to cells that synthesized proteins such as α-lactalbumin/lactoferrin/β-casein, albumin, and insulin, which are specific to lactocytes, hepatocytes, and islet cells, respectively.

However, breastmilk cells injected subcutaneously in immunodeficient mice did not form tumors. Subpopulations of hESCs and hiPSCs form teratoma-like masses when injected subcutaneously in immunodeficient SCID mice, an assay that is used as an indicator of in vivo pluripotency [36, 41]. Nevertheless, pluripotent epiblast stem cell subpopulations and adult cells with pluripotent features (very small embryonic-like cells (VSELs) and multilineage-differentiating stress-enduring cells (MUSE cells)) have been previously described failing to form teratomas when injected subcutaneously in SCID mice [7, 42, 43]. These reports demonstrate that not all pluripotent cells form teratomas under these conditions [7]. Despite that, these nontumorigenic pluripotent cells integrate into damaged tissues when transplanted into immunodeficient mice by local or i.v. injection [7, 43]. Future work is needed to determine whether the multilineage-differentiating hBSC populations described here will similarly integrate into damaged tissues.

**Figure 5:**
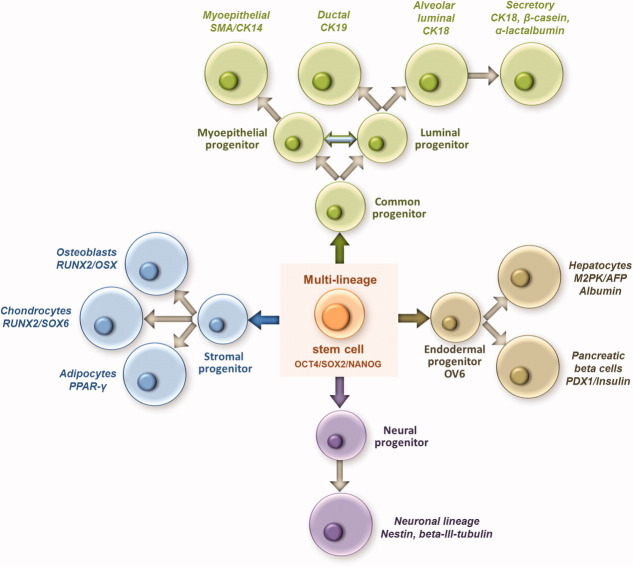
Proposed multilineage potential of breastmilk stem cells. Upregulation of the transcription factors (TFs) OCT4, SOX2, and NANOG during pregnancy and lactation results in broader differentiation potential of a mammary stem cell population under specific microenvironmental cues. Accessed via breastmilk, these cells can differentiate in corresponding microenvironments into not only the two main mammary epithelial lineages (green) but also into mesodermal lineages (blue), endodermal lineages (beige), and the neuroectodermal lineage (purple).

In the normal lactating breast, epigenetic modifications of the ESC-like cells identified here may be responsible for maintaining a controlled state of self-renewal, preventing uncontrolled proliferation and tumor formation. Similar theories have been previously suggested for other adult cells with pluripotent features (VSELs and MUSE cells) [7, 43]. We propose that the self-renewal TF network upregulated in the normal breast during pregnancy/lactation plays a fundamental role in the remodeling of the breast necessary to support its development toward a mature milk-secretory organ. We hypothesize that the underlying inherent expression of this pluripotency TF network might provide a priming epigenetic landscape to these cells to perform multilineage differentiation in vitro in tissue-specific microenvironments (Fig. 5). Importantly, disruption of this transcriptional network during pregnancy and lactation, failure to silence these genes during involution, or their aberrant imbalanced upregulation in the resting breast may be at the core of malignant transformation in the breast. In accordance with this, we have shown that forced ectopic expression of OCT4 in cells from the resting epithelium results in aberrant expansion of MaSCs possessing multilineage potential and displaying tumor-initiating features [33], which was in agreement with Tai et al. [27]. Future work will utilize hBSCs as a model to study molecular determinants of breast cancer associated with deregulation of their self-renewal machinery.

The multilineage differentiation potential of hBSCs and the lack of tumor formation in recipient animals suggest the potential use of hBSCs as a therapeutic alternative to hESCs and hiPSCs, the use of which is hindered by ethical and safety issues [36, 44, 45]. Because of its ethical, noninvasive and plentiful nature, breastmilk offers a novel resource of patient-specific stem cells for applications in regenerative medicine. Future work will examine the potential of tissue-specific transplantation and regeneration of damaged/diseased organs using hBSCs. In particular, the capacity of hBSCs to differentiate into insulin-producing cells may hold great promise for the development of personalized treatments for diabetes.

The natural presence in breastmilk of stem cells with multilineage differentiation potential also raises the question of the role of these cells during early infant development. Stem cell exchange between the mother and the embryo occurs in utero, resulting in microchimerism [46]. This may continue postnatally via breastfeeding. Indeed, this has been previously demonstrated for breastmilk leukocytes, which can cross the digestive epithelium and enter the infant's systemic circulation [47, 48]. It is proposed that similar to breastmilk leukocytes, hBSCs may also enter the systemic circulation and contribute to tissue homeostasis, repair, and/or regeneration in the infant. Future research should elucidate the role of these cells for the breastfed infant, generating implications for public policy related to early infant nutrition.

## CONCLUSIONS

A novel stem cell population was identified in the human lactating mammary gland and accessed noninvasively via breastmilk. We show that this cell population expresses ESC TFs ex vivo, expands via upregulation of these genes in 3D spheroid culture, and is capable of differentiating toward cells not only of the mammary lineage, but also of mesodermal and endodermal lineages. In the microenvironment of the breast, upregulation of self-renewal ESC TFs is likely to play a fundamental role in the remodeling of the mammary gland during pregnancy and lactation toward a milk-secretory organ. Importantly, deregulation of this self-renewal TF network might be at the core of invasive breast malignancies. Future work will establish the usefulness of breastmilk cells as models in breast cancer research and in regenerative medicine. Finally, the potential role of these cells for the breastfed infant merits further investigation.

## Supplementary material

Supplemental Figure S1. Ex vivo FACS analysis of ESC gene expression by freshly isolated breastmilk cells (n=12). (a) Gating strategy used to analyse FACS protein expression by breastmilk cells. A first gate was applied in the Forward versus Side Scatter plot as shown. In this gate, only the nucleated cells were selected via PI staining. The gated nucleated cell population was then separated into dead and viable cells, and only the latter were analysed for marker expression. (b) Distribution of the standardised differences in mean fluorescence intensity relative to controls (SDT-C) for ESC genes.

Supplemental Figure S2. Ex vivo co-expression of ESC genes by single freshly isolated breastmilk cells. (a) Double immunostaining of single cells demonstrating co-expression of ESC genes. Nuclei were stained with DAPI (blue). Scale bars: 5 μm. (b) FACS analysis of triple-stained cells co-expressing OCT4, SOX2 and NANOG.

Supplemental Figure S3. Quantitative real-time PCR assay for expression of additional ESC genes by breastmilk cells and comparison with human fibroblasts and hESCs. Expression of hTERT, REX1 and GDF3 is shown for 12-13 freshly isolated breastmilk cell samples from different participants (S1-S13). For hTERT and REX1, expression of spheroids at day 9 of growth (D9) is also shown, with a marked upregulation of mRNA expression under spheroid conditions. The great variability in mRNA expression between individuals is noted. Individual PCR reactions were normalized against internal controls (GAPDH) and plotted relative to the expression level of the fibroblasts. Bars represent the mean ± SEM.

Supplemental Figure S4. Breastmilk-derived cells display ESC-like traits in the absence of MEFs in 2D and 3D growth conditions, in contrast to mammary epithelial cultures derived from mammoplastic reductions of resting breast tissue. (a) Adherent ESC-like colonies of breastmilk cells grown in the absence of MEFs. Phase contrast micrograph of a colony with ESC morphology. Immunostaining of these colonies for OCT4 expression. (b) Mammary epithelial cell cultures derived from mammoplastic reductions of resting breast tissue did not express ESC genes. (c) Various sizes and morphologies of spheroids were obtained from 3D culture of hBSCs. Spheroids in passage 0-2 are shown. (d) Co-expression of ESC genes by single spheroid cells. Nuclei were stained with DAPI (blue). Scale bars: 20 μm.

Supplemental Figure S5. Additional examples of time-course of OCT4 expression by breastmilk cells in 3D culture conditions. Quantitative real-time PCR assay for expression of OCT4 in 13 breastmilk-derived cell samples (S1-S13) from 13 participants at day zero (D0 – fresh breastmilk cells prior to culture) and up to day 9 (D9) of spheroid growth. Each sample is identified by a different colour. Fibroblasts and hESCs are shown in black. Each time point was assayed in triplicate or duplicate depending on the number of cells available, and each bar represents the mean ± SEM. Samples S9- S13 are shown only for one time point due to inadequate material to run more time points. Individual PCR reactions were normalized against internal controls (GAPDH) and plotted relative to the expression level of human fibroblasts. Depending on the sample, a variable response of OCT4 expression to 3D culture was seen, with some samples that upregulated OCT4 expression to levels significantly higher than those of hESCs (e.g. S6, S12), and others that either increased OCT4 expression marginally (e.g. S3) or increased it to levels closer to those of hESCs (e.g. S5, S8, S9, S11).

Supplemental Figure S6. Spontaneous differentiation of hBSCs into cells from the three germ layers. (a-h) Phase Contrast micrographs of differentiated breastmilk-derived adherent colonies on day 14-21 of culture: (a) epithelial cells; (b) myoepithelial cells; (c) neuron-like cells; (d) fibroblast-like, stromal cells; (e) cobblestone-like cells; (f-h) mixed cell types. (i) Immunostaining of mixed mammary colonies (CK14+/CK18+), stromal cells (Vimentin, STRO-1), myoblast-like cells (desmin, SMA), endodermal colonies (α-fetoprotein - AFP, OV6), and neuron-like cells (β-III-tubulin). Nuclei were stained with DAPI (blue). Scale bars: 50 μm.

Supplemental Figure S7. Formation of fat globules within the cells under mammary differentiation conditions of hBSCs, suggesting milk production. (a-c) Phase Contrast micrographs of mammary colonies. Inserts show higher magnifications of cells with lipid droplets. Scale bars: 50 μm.

Supplemental Figure S8. Directed differentiation of hBSCs in 3D conditions. (a) Immunostaining of β-casein in mammospheres grown under mammary differentiation conditions. (b) Immunostaining of insulin in pancreatospheres grown under pancreatic differentiation conditions. (c) Immunostaining of cardiac T troponin in cardiospheres grown under cardiomyocyte differentiation conditions. Nuclei were stained with Hoechst 33342 (blue). Scale bars: 20 μm.
