# Supplementary figures and images for: Breastmilk Is a Novel Source of Stem Cells with Multilineage Differentiation Potential
Source: Stem Cells. 2012 Aug 3;30(10):2164–74. doi: 10.1002/stem.1188 (PMC3468727; doi:10.1002/stem.1188)

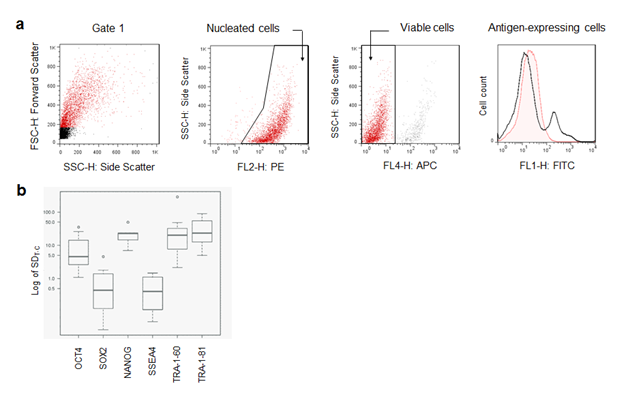

Supplement: Supplementary file 1 [file stem0030-2164-SD1.tif]

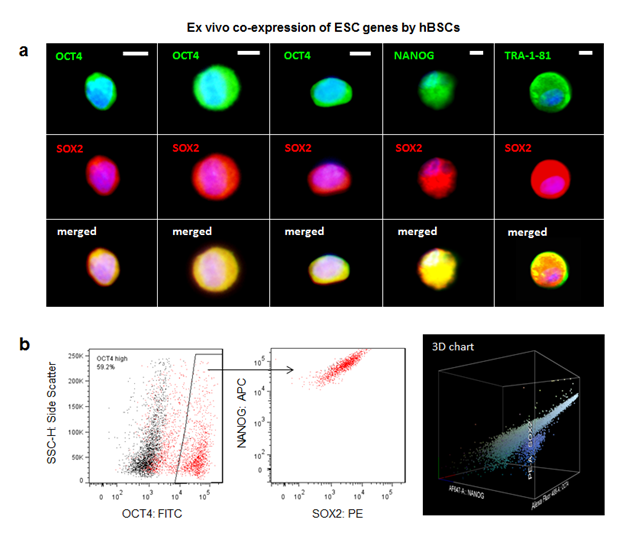

Supplement: Supplementary file 2 [file stem0030-2164-SD2.tif]

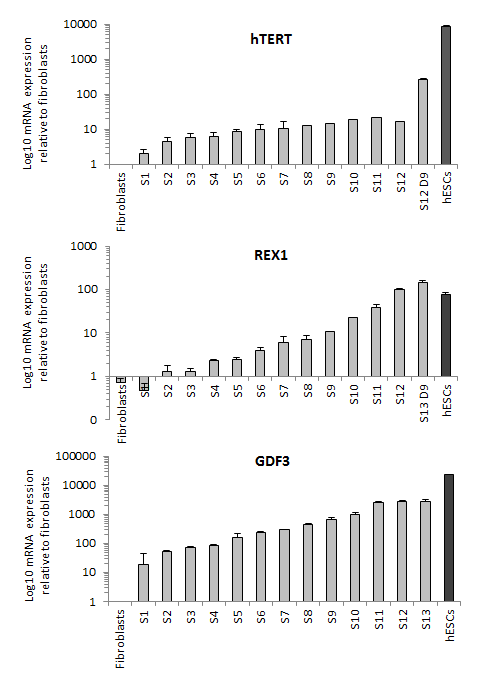

Supplement: Supplementary file 3 [file stem0030-2164-SD3.tif]

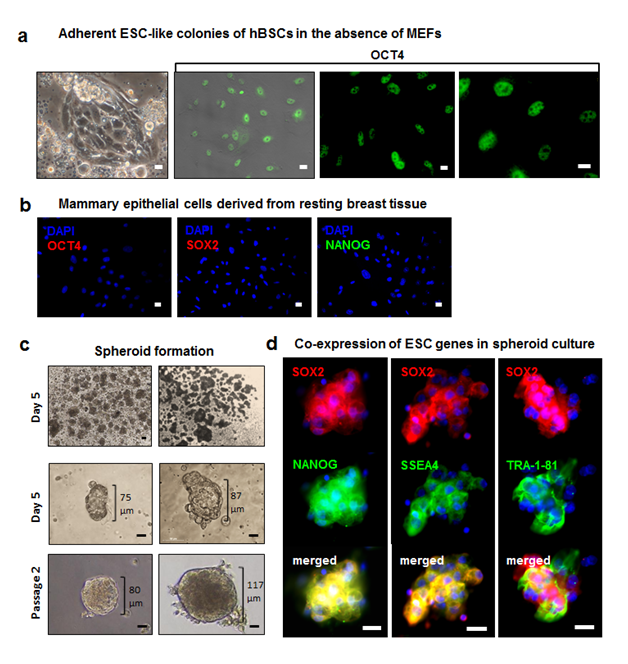

Supplement: Supplementary file 4 [file stem0030-2164-SD4.tif]

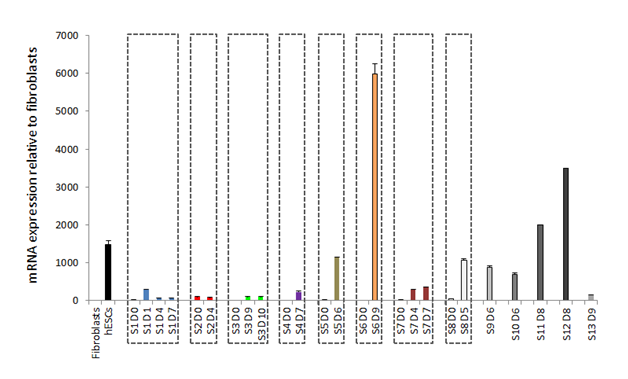

Supplement: Supplementary file 5 [file stem0030-2164-SD5.tif]

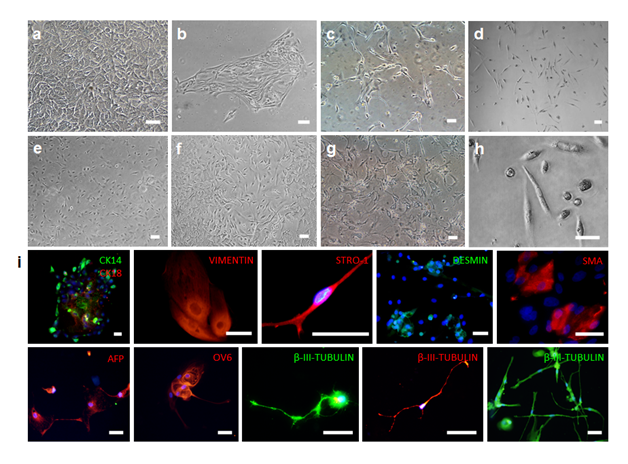

Supplement: Supplementary file 6 [file stem0030-2164-SD6.tif]

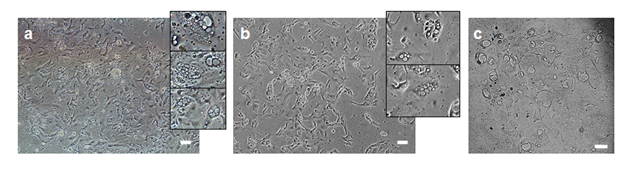

Supplement: Supplementary file 7 [file stem0030-2164-SD7.tif]

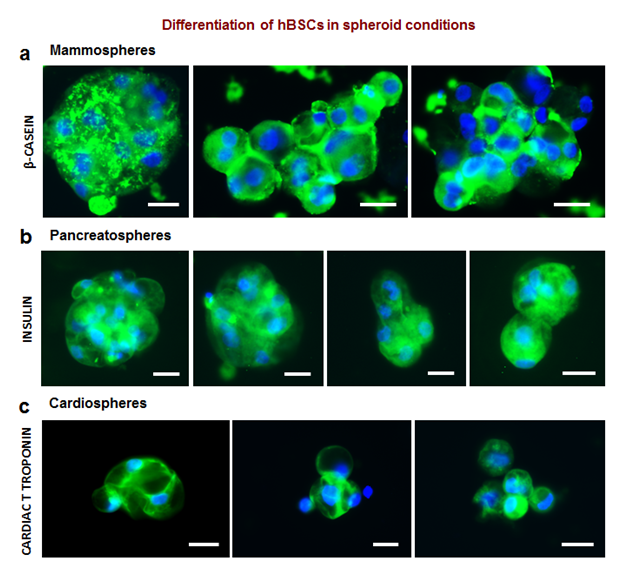

Supplement: Supplementary file 8 [file stem0030-2164-SD8.tif]
