# Supplementary material for: Breastmilk Is a Novel Source of Stem Cells with Multilineage Differentiation Potential
Source: Stem Cells. 2012 Aug 3;30(10):2164–74. doi: 10.1002/stem.1188 (PMC3468727; doi:10.1002/stem.1188)
Supplement: Supplementary file 9 [file stem0030-2164-SD9.pdf]

SUPPLEMENTAL INFORMATION

Supplemental Table S1. Markers tested and corresponding antibodies used.

Marker	Company	Cat. No	Applications used
Oct4	Miltenyi (Stemgent)	130-095-635	FACS (1:100), IF (1:100), IHC (1:100)
Oct4A	Santa Cruz Biotech.	sc-5279	FACS (1:50), IF (1:100), IHC (1:100)
Oct4	Abcam	ab18976	IF (1:100)
Oct4A	Cell Signalling Tech.	2840	WB (1:1000)
Sox2	Miltenyi (Stemgent)	130-095-636	FACS (1:50), IF (1:50), IHC (1:50)
Sox2	Cell Signalling Tech.	3579	IF (1:100), WB (1:1000)
Nanog	Santa Cruz Biotech.	sc-33759	FACS (1:100), IF (1:100), IHC (1:100)
Nanog	Abcam	ab80892	IF (1:100)
Nanog	R&D Systems	AF1997	IF (1:50)
Nanog	Cell Signalling Tech.	3580	WB (1:1000)
SSEA4	Miltenyi (Stemgent)	130-095-622	FACS (1:100), IF (1:100), IHC (1:100)
SSEA4	Hybridoma cell line*	MC-813-70	IF (1:30)
Tra-1-60	Miltenyi (Stemgent)	130-095-625	FACS (1:300), IF (1:300), IHC (1:300)
Tra-1-60	ABCAM	ab16288	IF (1:100)
Tra-1-81	Miltenyi (Stemgent)	130-095-626	FACS (1:300), IF (1:300), IHC (1:300)
Tra-1-81	ABCAM	ab16289	IF (1:100)
β-actin	Sigma-Aldrich	A5441	WB (1:1000)
CK14	AbD Serotec	SEMCA890HT	IF (1:100)
α-SMA	Sigma-Aldrich	A2547-0.2ML	IHC (1:100)
CK18	Abcam	ab32118	IF (1:100)
β-casein	AbD Serotec	SE18602655	IF (1:20)
β-casein	Santa Cruz Biotech.	sc-53189	IF (1:100)
α-lactalbumin	Dako	A057901	WB (1:500)
lactoferrin	MP Biomedicals	670581	WB (1:500)
β-III-tubulin	Covance	PRB-435P	IF (1:1000)
Nestin	Miltenyi (Stemgent)	130-095-648	IF (1:100)
OV6	R&D Systems	MAB2020	IF (1:200)
M2PK	Cell Signalling Tech.	3198	IF (1:200)
α-Fetoprotein (AFP)	ZYMED Laboratories	18-0055	IF (1:200)
Albumin	Sigma-Aldrich	A-3293	IF (1:200)
PDX1	Santa Cruz Biotech.	sc-14662	IF (1:100)
PDX1	Cell Signalling Tech.	2437S	IF (1:100)
Insulin	Santa Cruz Biotech.	sc-52040	IF (1:100)
Insulin	Cell Signalling Tech.	4590S	IF (1:100)
RUNX2	Santa Cruz Biotech.	sc-101145	IF (1:100)
OSX	Santa Cruz Biotech.	sc-133871	IF (1:100)
Sox6	Santa Cruz Biotech.	sc-20092	IF (1:100)
PPAR-γ	Santa Cruz Biotech.	sc-1984	IF (1:100)
Vimentin	Sigma-Aldrich	V5255	IF (1:100)
STRO-1	Hybridoma cell line**		IF (Culture supernatant)
Desmin	Sigma-Aldrich	D1033-0.2ML	IF (1:100)
Cardiac T troponin	Abcam	ab45932	IF (1:100)

FACS: Fluorescence Activated Cell Sorting; IF: Immunofluorescence staining; IHC: Immunohistochemistry; WB: Western Blotting. *Culture supernatant was purchased from the Development Studies Hybridoma Bank. **The hybridoma cell line was purchased from the Development Studies Hybridoma Bank.
