# Supplementary material for: Breastmilk Is a Novel Source of Stem Cells with Multilineage Differentiation Potential
Source: Stem Cells. 2012 Aug 3;30(10):2164–74. doi: 10.1002/stem.1188 (PMC3468727; doi:10.1002/stem.1188)
Supplement: Supplementary file 10 [file stem0030-2164-SD10.pdf]

Supplemental Table S2. Probes used for RT-PCR (Taqman, Applied Biosystems).

Gene	Applied Biosystems Reference Number
HuGAPDH(20x)	4352934E
OCT4 (POU5F1)	Hs03005111_g1
SOX2	Hs01053049_s1
NANOG	Hs02387400_g1
KLF4	Hs00358836_M1
hTERT	Hs00972656_m1
REX1 (REXO1)	Hs00810654_m1
GDF3	Hs00220998_m1
