# Supplementary material for: Breastmilk Is a Novel Source of Stem Cells with Multilineage Differentiation Potential
Source: Stem Cells. 2012 Aug 3;30(10):2164–74. doi: 10.1002/stem.1188 (PMC3468727; doi:10.1002/stem.1188)
Supplement: Supplementary file 11 [file stem0030-2164-SD11.pdf]

Supplemental Table S3. ***Ex vivo*** **ESC gene expression levels in breastmilk cells analysed by FACS (n=12).** SD_{T-C} denotes the standardised difference in Mean Fluorescence Intensity (MFI) between the control and the test. It is noted that among the 12 subjects analysed for each gene, a shift in the stained sample compared to the negative control was observed in some cases. In these cases, it is not possible to record the percentage positive population and therefore, the range of % expression (fourth column) includes only the samples where a clear positive population was obtained. The last column reports the number of subjects out of 12 where a shifted positive population was obtained. (*n=5 for NANOG)

**Marker**	**Range of SD_{T-C}**	**Mean SD_{T-C} (±SEM)**	**Range of % expression**	**Shifted positive**
OCT4	1.1-35.5	9.7±3.2	9.3-87.9	2
SOX2	0.0-4.6	1.0±0.4	4.3-73.6	8
NANOG*	7.0-49.8	23.6±7.2	37.4-99.5	0
SSEA4	0.1-1.5	0.6±0.2	0.1-27.0	3
TRA-1-60	2.1-287.5	41.9±22.7	14.4-93.0	5
TRA-1-81	4.9-90.5	32.7±8.1	13.1-91.9	4
