# Supplementary material for: Breastmilk Is a Novel Source of Stem Cells with Multilineage Differentiation Potential
Source: Stem Cells. 2012 Aug 3;30(10):2164–74. doi: 10.1002/stem.1188 (PMC3468727; doi:10.1002/stem.1188)
Supplement: Supplementary file 12 [file stem0030-2164-SD12.pdf]

Supplemental Table S4. Analysis of ESC-like and non-ESC-like colony formation potential by hBSCs in 2D feeder culture under hESC culture conditions. Each sample represents a breastmilk sample from a different breastfeeding mother (n=11). For each sample, analysis was done in a well of a 6-well plate at day 10 of growth. The % of ESC-like and non-ESC like adherent colonies is reported as % out of total colonies observed in each well for each sample. Although all ESC-like colonies expressed OCT4, most non-ESC-like colonies did not express OCT4, or expressed it at low levels or heterogeneously compared to ESC-like colonies. It is noted that single adherent cells that failed to expand but expressed ESC genes were observed and were not included in this analysis. Moreover, in all cases numerous spheroids formed in suspension in these cultures above the adherent cells, and these were also not included in this analysis.

Sample ID	Total colonies	Number of ESC-like colonies	Number of non-ESC-like colonies	% ESC-like colonies	% non-ESC-like colonies	% ESC-like colonies expressing OCT4	% non-ESC-like colonies expressing OCT4
S1	16	14	2	87.5	12.5	100.0	50.0
S2	10	9	1	90.0	10.0	100.0	0.0
S3	5	5	0	100.0	0.0	100.0	-
S4	9	8	1	88.9	11.1	100.0	0.0
S5	14	12	2	85.7	14.3	100.0	0.0
S6	4	4	0	100.0	0.0	100.0	-
S7	5	5	0	100.0	0.0	100.0	-
S8	3	3	0	100.0	0.0	100.0	-
S9	76	52	24	68.4	31.6	100.0	16.7
S10	16	14	2	87.5	12.5	100.0	50.0
S11	433	333	100	76.9	23.1	100.0	24.0
